# The effects of low concentrations of the enantiomers of mushroom alcohol (1-octen-3-ol) on *Arabidopsis thaliana*

**DOI:** 10.1080/21501203.2014.902401

**Published:** 2014-04-04

**Authors:** Richard Hung, Samantha Lee, Joan W. Bennett

**Affiliations:** Department of Plant Biology and Pathology, Rutgers, The State University of New Jersey, 59 Dudley Rd., New Brunswick, 08901, NJ, USA

**Keywords:** volatile organic compound, mushroom alcohol, 1-octen-3-ol, semiochemical, *Arabidopsis thaliana*, seed germination

## Abstract

“Mushroom alcohol,” or 1-octen-3-ol, is a common fungal volatile organic compound (VOC) that has been studied for its flavor properties, its effects on fungal spore germination, mushroom development, and as a signaling agent for insects. Far less is known about its effects on plants. We exposed *Arabidopsis thaliana* seeds, under conditions conducive to germination, to high (10 and 100 mg/1) and low concentrations (1, 2, and 3 mg/1) of racemic, *S*, and *R* forms of 1-octen-3-ol for 3 days. In addition, 1-, 2-, 3-, and 4-week-old *A.* *thaliana* plants also were exposed to 1 mg/1 of the compounds for the same period of time. Seedling formation was retarded with all tested levels of exposure to 1-octen-3-ol for both enantiomers and the racemer, while 95% of unexposed control seeds germinated to seedling within 3 days. There was a dose-dependent response in the reduction of seedling formation between 1 mg/1 and 3 mg/1 of exposure. When exposed seeds were removed from the VOC, nearly all resumed germination. Young plants exposed to 1 mg/1 of the *R* and *S* enantiomers of 1-octen-3-ol exhibited a mild inhibition of growth and chlorophyll production at 2 and 3 weeks but not at 4 weeks.

## Introduction

Volatile organic compounds (VOCs) are organic compounds capable of entering the gas phase under conditions of normal atmospheric temperature and pressure. “Mushroom alcohol,” or 1-octen-3-ol, is an 8-carbon alcohol produced by the enzymatic oxidation and cleavage of linoleic acid (Wurzenberger and Grosch 1984). It is one of the most abundant VOCs produced by fungi and is characteristic of fungal aromas and flavors (Mau et al. 1992, 1997; Venkateshwarlu et al. 1999). It has two optically active isomers. The *R*-(−)-1-octen-3-ol form has a more mushroom like odor while the (*S*)-(+)-l-ocen-3-ol form is also mushroom-like with a moldy-grass like note (Mosandl et al. 1986). The compound has a low odor threshold and can be detected at levels of 0.0001 mg/1 in water (Wnuk et al. 1983). In addition to fungi, this VOC is detected from a widespread group of animals and plants (Bernier et al. 2000; Ramoni et al. 2001; Maggi et al. 2010). In insects, it functions as a signaling molecule (semiochemical), especially in mediating host location cues for flies, mosquitoes, and mites where it orients biting flies and other blood-sucking arthropods to their hosts (Luntz 2001). Along with CO_2_ it is the compound that attracts malaria mosquitoes and can be used as a bait in mosquito traps (Nilssen 1998). In addition to its flavor and arthropod signaling properties, 1-octen-3-ol has been used as an indicator of fungal spoilage in stored grains (Tuma et al. 1989; Schnürer et al. 1999); functions as a self-inhibitor of spore germination in *Penicillium paneum* (Chitarra et al. 2005); delays the formation of fruiting bodies in *Agaricus bisporus* (Noble et al. 2009); and inhibits the radial growth of microfungi from several genera (Okull et al. 2003; Chitarra et al. 2004). It also has been used to control *Lecanicillium fungicola*, cause of bubble disease in white button mushroom (Berendsen et al. 2013). The literature on the biological activity of 1-octen-3-ol is widely scattered and isolated by discipline; perhaps the most comprehensive single review on its broad range of biological activities is by Combet et al. (2006).

Plant growth-promoting rhizobacteria emit a number of VOCs that have beneficial growth effects on their plant hosts (Ryu et al. 2003; Vespermann et al. 2007; Zhang et al. 2008; Xie et al. 2009). Similarly, it has been postulated that biocontrol agents such as *Pseudomonas* and *Trichoderma* may evoke their beneficial effects on plant growth through a variety of mechanisms that may include VOCs (Cook 1993; Howell 2003; Weller 2007; Hung et al. 2013). Nevertheless, compared to bacterial VOCs, far less is known about the effects of fungal VOCs on germination efficiency, seedling formation, and plant health.

At high concentrations (130 mg/1), Splivallo et al. (2007) demonstrated that 1-octen-3-ol inhibited root growth and lowered chlorophyll concentration in *Arabidopsis thaliana.* At a low concentration (10 µl of 0.1 M), Kishimoto et al. (2007) showed that 1-octen-3-ol enhanced resistance of mature *A. thaliana* to *Botrytis cinerea* and activated some of the same defense genes turned on by ethylene and jasmonic acid signaling. Both studies employed the racemic version of 1-octen-3-ol and diluted it in commercial solvents. Since chiral discrimination plays a central role in the activity of many biosystems (He and Beesley 2005) and since many commercial solvents have an adverse effect on plant growth, we have examined the effect of these parameters using *A. thaliana* as our test system. The goal of this research was to determine if low concentrations (1–3 mg/1) of the racemic, (*S*)-(+)-l-ocen-3-ol, and *R*-(−)-1-octen-3-ol forms of mushroom alcohol had different effects on *A. thaliana* seed germination, seedling formation, and plant growth in the absence of commercial solvents.

## Materials and methods

### Plant material and seed preparation

*Arabidopsis thaliana* seeds (ecotype Columbia 7) were obtained from Dr Thomas Leustek of the Rutgers University Department of Plant Biology and Pathology, New Brunswick, NJ, USA. Surface-sterilized seeds were sown on Murashige & Skoog (MS) media with vitamins, 3% sucrose, and 0.3% Gellan Gum Powder (G 434 Phyto Technology Laboratories, Shawnee Mission, KS, USA). Seeds used in seedling-formation studies were sown on Petri dishes with 20 ml MS media, 80 seeds per plate. The sown plates were placed at 4°C in the dark for 3 days to stratify the seeds. Seeds used to grow plants for exposure assays were sown individually in test tubes with plant-tissue culture caps. The test tubes with seeds were then stratified as described above. After 3 days, stratified seeds were placed in a growth chamber with the following conditions: 21°C ± 2°C and 16 h photoperiod. After a 72 h exposure, culture vessels and plants were removed for destructive testing. The experiment was performed in quadruplicate.

### Chemicals and exposure conditions

Stratified seeds in Petri dishes were exposed to different concentrations of vaporized 1-octen-3-ol, or to air alone, for a 3-day test period. The racemic form of 1-octen-3-ol was purchased from Sigma-Aldrich (05284-25G). The enantiomers (*S*)-(+)-l-ocen-3-ol and *R*-(−)-1-octen-3-ol were gifts from Bedoukian Research, Danbury, CT, USA. In our germination experiments, three concentration of 1-octen-3-ol were used: 1, 2, and 3 mg/1. In some preliminary studies, seeds were also exposed to 10 and 100 mg/1 of these VOCs. As positive controls, chloroform and dichloromethane were tested at 3 mg/1. Each Petri plate containing stratified seeds was placed individually into a 1-1 plant culture vessel with a natural polypropylene closure (C579, PhytoTechnology Laboratories). The desired concentrations of VOCs were obtained by the addition of an aliquot of undiluted liquid compound calculated to deliver the correct concentration. The liquid was deposited on to the surface of the glass on the top third of the vessel along the inside of the vessel. The vessel was sealed to contain the VOC for the duration of the experiment and then placed on a 1 inch throw rotator at 40 rpm in order to volatilize and evenly distribute the compound, in an incubator at 21°C ± 2°C in the dark. The vegetative plants were exposed in a similar manner with the following changes. Each 1 1 plant culture vessel contained four plants and exposed to a 16-h photo period. The vegetative plants were exposed to 1 mg/1 of undiluted liquid compounds at 1, 2, 3, and 4 weeks of age. The exposure time was for 3 days. Plants were harvested for destructive testing at 10, 17, 24, and 31 days.

### Scoring germination stages

**Figure 1.**
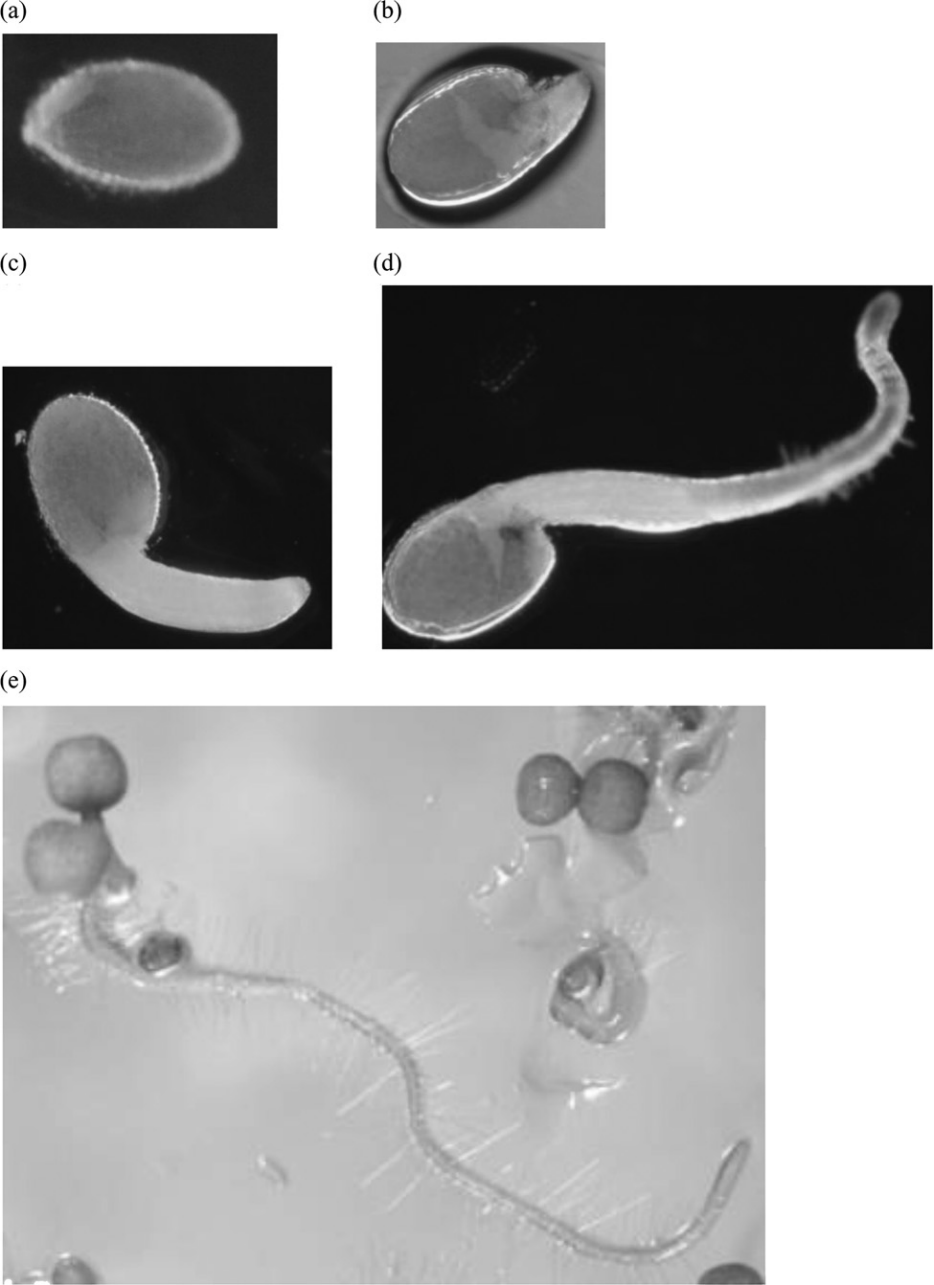
Stages of seedling formation in *A. thaliana.* (a) No germination (Stage 1). (b) Germination: broken seed coat (Stage 2). (c) Radicle emergence: <1 mm (Stage 3). (d) Extended radicle: >1 mm (Stage 4). (e) Full seedling formation: green cotyledons, long root (Stage 5).

The seeds were exposed for 72 hours and then removed from the culture vessels, examined under a binocular microscope, and scored into five germination-to-seedling stages as follows: 1 = no visible germination; seed coat (testa) intact; 2 *—* initial testa rupture; 3 = radicle emergence (<1 mm); 4: extended radicle (>1 mm); 5 = cotyledon visible; complete germination to seeding (see Figure 1).

### Plant mass and chlorophyll concentration

After exposure to 1 mg/1 1-octen-3-ol, plants were removed from the test conditions, the roots were removed, and the above ground portion of the plant was weighed to facilitate the analysis of the chlorophyll data which is expressed in relation to the fresh weight of the plant. The excised roots were then weighed independently. The total chlorophyll concentration of individual plants was obtained using the method developed by Jing et al. (2002) with some modifications. The chlorophyll concentration measurements were determined using a spectrophotometer (DU800, Beckman Coulter, Brea, CA, USA) and acetone extracts of whole rosettes. The chlorophyll was extracted using 80% acetone and each solvent extract contained one plant per treatment. The plants were soaked overnight at 4°C in darkness prior to obtaining photometric readings at absorbance 663 and 645 nm. The total chlorophyll concentration (chlorophyll a and b) was determined with the following equation, (8.02*A663 + 20.2*A645)*V/1000*W (Palta 1990).

The data was analyzed and plotted using Excel software (Microsoft, Redmond, WA, USA) and SigmaPlot (SPSS Science Inc., Chicago, IL, USA). To test the significance of the exposure studies, ANOVA was performed with the aggregated data. The seedling formation tests were performed four times for a total of 320 seeds tested per condition (control, 1, 2, and 3 mg/1). There were two replicate containers for each vegetative plant exposure experiment for a total of eight plants per experiment per condition. The experiments were performed four times for a total of 32 vegetative plants tested for each compound at each time period.

## Results

### Exposure concentrations

In preliminary seedling-formation tests at 10 and 100 mg/1 of 1-octen-3-ol for 3 days, there were no visible indications of germination, that is, no seeds reached Stage 2. All subsequent tests were done at the lower concentrations. Preliminary tests on exposure of 2-week-old plants at concentrations of 2 mg/1 and above caused death before the end of the exposure period (3 days). All subsequent experiments were done at 1 mg/1.

### Germination studies

**Figure 2.**
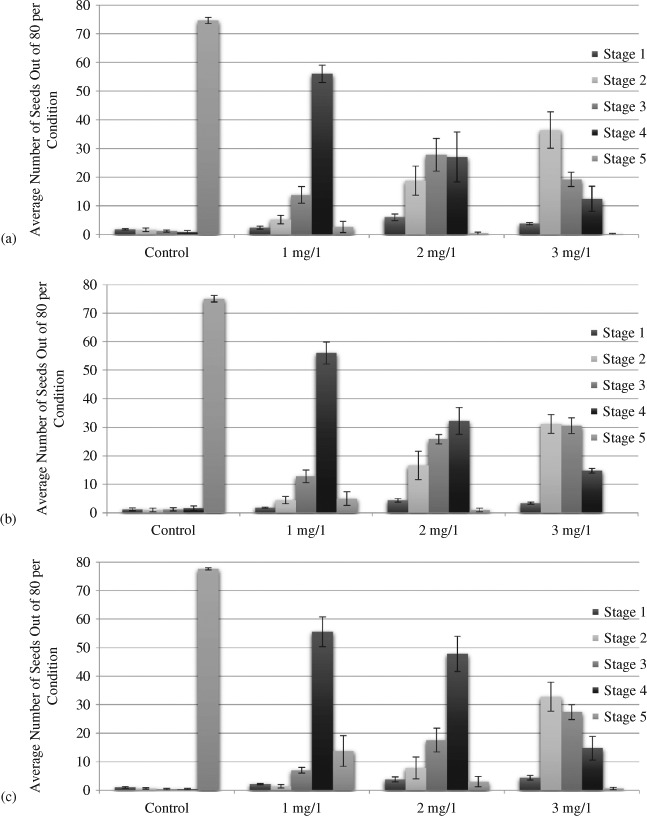
Effect of O, 1, 2, and 3 ppm of 1-octen-3-ol for 3 days on seedling formation in *A. thaliana.* (a) Average number of seeds that have reached each stage when exposed to racemic 1-octen-3-ol. (b) Average number of seeds that have reached each stage when exposed to *R’* 1-octen-3-ol. (c) Average number of seeds that have reached each stage when exposed to *S’* 1-octen-3-ol. Error bars represent standard error of the mean, ANO *p* < 0.001 for all stages.

Seed germination after exposure of racemic, *R*, and *S* forms of 1-octen-3-ol at 0, 1, 2, and 3 mg/1 for 3 days is shown in Figure 2. Between 93% and 97% of control seeds germinated and formed seedlings (Stage 5). Similarly, in solvent controls, at 3 mg/1, 95% of seeds exposed to dichloromethane and 94% of seeds exposed to chloroform reached Stage 5 (data not shown).

In contrast, seedling formation was retarded at all three levels of exposure to all three forms of 1-octen-3-ol. When seeds were treated with 1 mg/1 racemic 1-octen-3-ol, only 3% completed germination to seedling; while even fewer (0.25%) seeds reached this stage with exposure to 2 and 3 mg/1 (Figure 2(a)). In general, there was a dose-dependent response with increasing retardation of seed germination and seedling formation with higher levels of volatile exposure. With the exception of 1 mg/1 of the *S* form, in which 17.4% of seeds reached Stage 5, and 1 mg/1 of the *R* form where 5% of seeds reached this stage, levels of retardation of seed germination for each of the two stereo isomers were similar to the racemic form (Figure 2(b, c)). No seeds reached Stage 5 in the presence of 3 mg/1 of the *R* form of 1-octen-3-ol (Figure 2(c)). A statistically (*p* = 0.0001) significant retardation of germination and radicle extension was obtained for 1, 2, and 3 mg/1 exposure for all three forms of 1-octen-3-ol.

Nevertheless, while seedling formation was retarded, seed germination was not inhibited at these concentrations. At 3 mg/1, almost half of the seeds had a broken seed coat and over half showed evidence of radicle emergence. Moreover, when removed from the presence of 1-octen-3-ol, the treated seeds completed seedling formation. Experiments at higher concentrations up to 100 mg/ 1 showed that treated seeds, although delayed in seedling formation in the presence of 1-octen-3-ol, when removed from the test conditions were able to recover and complete seedling formation at the same frequency as negative controls.

### Vegetative plants

**Figure 3.**
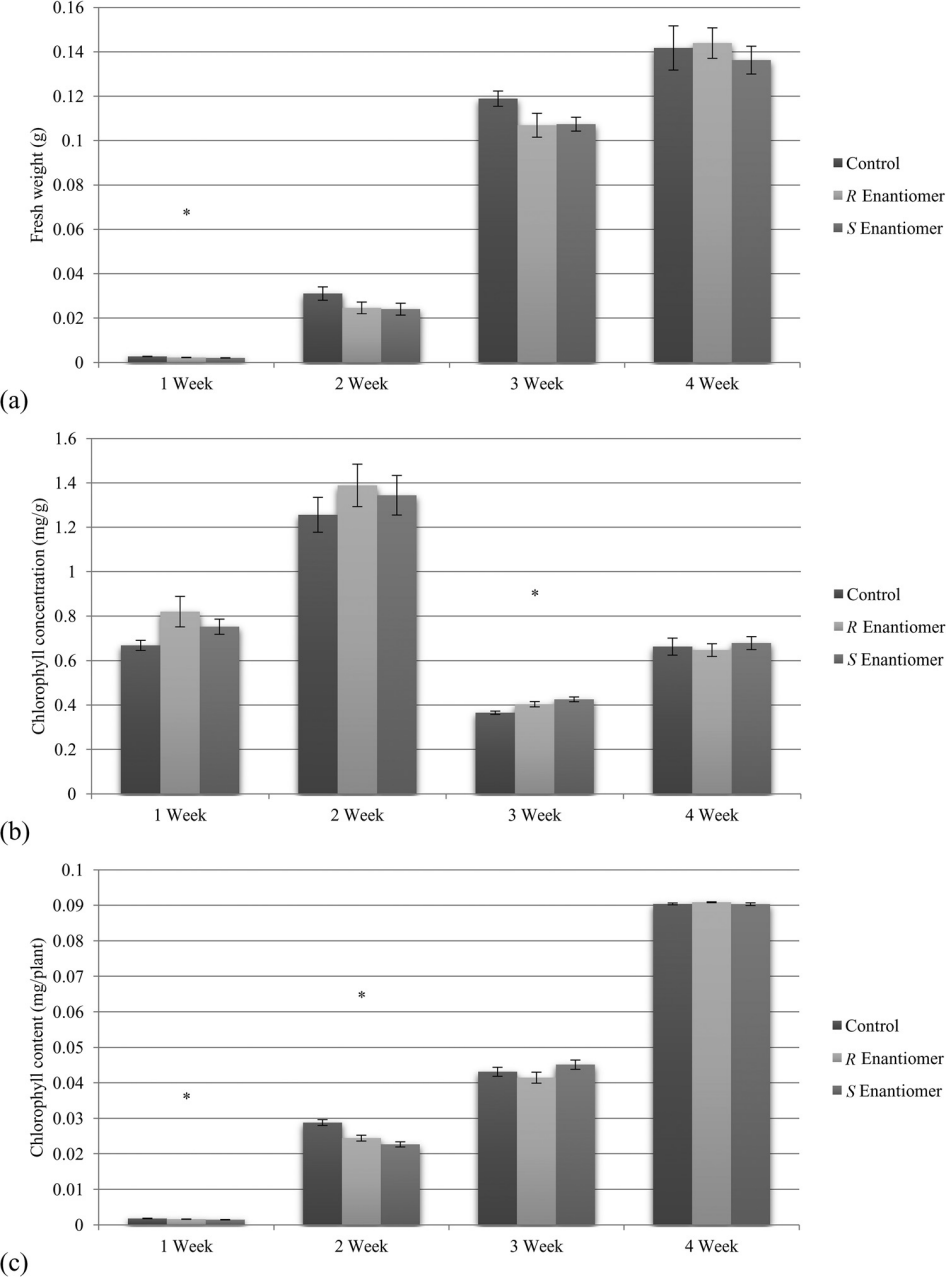
Effect of 3 day exposure at 0 and 1 ppm of 1-octen-3-ol enantiomers on 1-, 2-, 3-, and 4-week-old *A. thaliana.* (a) Comparison of fresh weight, (b) Comparison of chlorophyll concentration, (c) Comparison of chlorophyll content per plant. Error bars represent standard error of the mean, ANOVA *p* < 0.01 for all statistically significant data sets marked by an asterisk.

Exposure of young vegetative plants to 1-octen-3-ol *R* and *S* enantiomers at 1 mg/1 caused statistically significant decreases in plant fresh weight at 1, 2, and 3 weeks. (See Figure 3(a–c)). Decreased above- and below-ground biomass, were observed. In addition, there was a statistically significant decrease in chlorophyll content at 1 and 2 weeks but no differential effect at 3 and 4 weeks. There were two instances of chlorophyll concentration increase: the S enantiomer at 1 week and the R enantiomer at 3 weeks. Four-week-old *A. thaliana* plants exposed to the R enantiomer had an average increase of 0.47 mg of chlorophyll content as compared to controls. In both cases where chlorophyll-concentration increase was observed, the corresponding fresh weight was decreased, indicating that the chlorophyll-concentration increase was likely due to a smaller plant size and not increased chlorophyll content.

In conclusion, the *R* form of 1-octen-3-ol has a stronger effect than the *S* enantiomer in suppressing seedling formation, but in general, both forms of the compound retard, but do not suppress, seed formation At 1 mg/1, both the *R* and the *S* forms of mushroom alcohol retard growth for 1, 2, and 3-week-old plants. For 2 and 3-week-old plants, only the *R* form has a statistically significant inhibitory effect on chlorophyll content.

## Discussion

Plants and their seeds have evolved divergent responses to the environmental signals that involve adaptation to the prevailing environment. In addition to the basic requirements for water, oxygen, and appropriate temperature, plants also may be sensitive to factors such as light, nitrate, and signaling biomolecules. Some VOCs emitted by plants such as allyl isothiocyanate and methyl isothiocyanate play important roles in mediating allelo-pathic effects and as cues for the presence of proximate competitors (Vaughn and Boydston 1997; Kegge and Pierrk 2010). These factors interact and affect the ability of seeds to come out of dormancy (defined as the failure of an intact and viable seed to complete germination under favorable conditions (Bewley 1997) and of vegetative plants to grow properly. For example, foliage clipping in sagebrush yield VOCs that inhibit germination of neighboring seeds (Karban 2007) and VOCs emitted by snapdragon flowers inhibit root growth in *A. thaliana* seedlings (Horiuchi et al. 2007). Important flavor compounds such as those associated with the distinctive odors of sassafras, jasmine, and tobacco have bioregulatory properties that include both stimulation and inhibition of spores and seeds (French 1985; Leather and French 1990). Composts with high microbial populations suppress damping off but the specific mechanisms are not well understood (Craft and Nelson 1996).

Many agriculturally and environmentally important chemicals are chiral molecules and sometimes the enantiomers exhibit different biological effects (He and Beesley 2005). Most of the published literature on 1-octen-3-ol concerns either its properties as a mushroom flavor compound (Zawirska-Wojtasiak 2004) or its importance in attracting biting insects (Bernier et al. 2000; Luntz 2001). In both of these cases, the *R* enantiomer (“roctonal”) is the active component. On the other hand, either of the optically active versions of this alcohol exhibited attracting and molting activities in pine wood nematode (Matsumori et al. 1989), and the racemic form was effective in inhibiting fungal spore germination (Chitarra et al. 2004, 2005; Berendsen et al. 2013). In our studies, the germination of *A. thaliana* seeds exposed to racemic 1-octen-3-ol and its enantiomers was retarded. The *R* form was somewhat more active; however, the *S* form also exhibited significant inhibitory effects, especially on seed germination. We conclude that although chirality of 1-octen-3-ol is important to insect sensory perception and human flavor detection, it does not have a differential effect on inhibition of seedling formation in plants.

It is also important to note that seeds exposed to all three concentrations of 1-octen-3-ol tested were able to resume seedling formation once removed from the testing conditions. This shows that 1-octen-3-ol functions as a retardant, not a toxicant. As plant physiologists learn more about seed dormancy, there is increasing recognition that it is an active physiological state, with complex regulatory networks that integrate environmental signals to regulate germination stages (Finch-Savage and Leubner-Metzger 2006). The vegetative plants were much less susceptible to exposure to 1-octen-3-ol enantiomers. There were some detrimental effects up until week 3; however, by the fourth week, the plants were no longer affected by the volatile. The effects are most obvious at week 2 and 3. The enantiomers had a similar detrimental effect on fresh weight during these two periods but different effects on chlorophyll content. The effects observed suggest that 1-octen-3-ol affects plants during its rapid growth stage. Once the plant has matured and switches from vegetative to reproductive growth, the volatile no longer has a detectable effect on the plant.

In conclusion, at low concentrations both enantiomers of mushroom alcohol (1-octen-3-ol), a well-known odorant and semiochemical, retard seed germination, seedling formation, and vegetative growth in *Arabidopsis*, suggesting that other ecologically important aspects of VOC-mediated fungal-plant communications merit further study. *Arabidopsis* mutants affecting different stages of the seed dormancy response and hormone pathways are available, making this plant an excellent model for studying the interkingdom signaling activity of fungal VOCs in general and 1-octen-3-ol in particular. Further studies are underway in our laboratory.
